# Worker’s Health Quest: disease, prevention, and edutainment

**DOI:** 10.47626/1679-4435-2023-810

**Published:** 2023-04-18

**Authors:** Arthur Brito-Marcelino, Katienne Brito Marcelino, Eloanne Cerqueira

**Affiliations:** 1 Hospital de Urgência de Sergipe, Secretaria Estadual de Saúde de Sergipe, Aracaju, SE, Brazil; 2 Faculdade de Medicina, Universidade de Gurupi, Gurupi, TO, Brazil; 3 Graduação em Farmácia, Universidade Federal de Sergipe - Campus Lagarto, Lagarto, SE, Brazil

**Keywords:** occupational health, disease prevention, learning, occupational diseases, games and playthings, saúde do trabalhador, prevenção de doenças, aprendizagem, doenças profissionais, jogos e brinquedos

## Abstract

**Introduction:**

Edutainment can be used as a tool to promote health education.

**Objectives:**

To develop an activity from edutainment focusing on occupational health.

**Methods:**

A descriptive study based on literature review and game development through the following steps: research, development, construction, and final product.

**Results:**

A trail game was developed containing information on the following occupational diseases: noise-induced hearing loss, work-related voice disorder, pneumoconiosis, repetitive strain injury/work-related musculoskeletal disorders, occupational dermatosis, exposure to biological material, occupational stress, radiation exposure, SARS-CoV-2 infection, child labor, and exogenous poisoning (pesticides).

**Conclusions:**

Educational games can be useful in preventing occupational health problems and promoting quality of life.

## Abstract (pt)

**Introdução:**

O aspecto lúdico pode ser utilizado como ferramenta para promover a educação em saúde.

**Objetivos:**

Desenvolver atividade a partir de uma perspectiva lúdica e informativa com foco na saúde do trabalhador.

**Métodos:**

Estudo descritivo com base em uma revisão bibliográfica e no desenvolvimento de um jogo, seguindo as etapas de pesquisa, desenvolvimento, construção e produto final.

**Resultados:**

Foi construído um jogo de trilha contendo informações sobre os seguintes agravos ocupacionais: perda auditiva induzida por ruído, distúrbio vocal relacionado ao trabalho, pneumoconiose, lesão por esforço repetitivo/doença osteomuscular relacionada ao trabalho, dermatose ocupacional, acidente com material biológico, estresse ocupacional, exposição à radiação, infecção por COVID-19, trabalho infantil e intoxicação exógena por agrotóxicos.

**Conclusões:**

Os jogos e as brincadeiras educativas podem ser úteis nos cuidados de prevenção dos agravos à saúde do trabalhador e na promoção da qualidade de vida.

## INTRODUCTION

Certainly, one of the greatest forms of human expressiveness is through their work. Human beings change reality, promote subsistence, create social relationships, and build their own identity through their occupation. However, the same work which is a source of dignity is also a source of pain and disease for those who perform it.^1^

In a context of work overload, high work demands, and high competition, occupational diseases arise and threaten the health and well-being of many workers.^2^ According to a study conducted between 2009 and 2016 based on social security websites and the Classificação Nacional de Atividades Econômicas (CNAE, Brazilian National Classification of Economic Activities), 122,937 workers presented permanent disability and 21,490 died as a result of occupational outcomes.^3^

In order to minimize these occupational diseases, the Centros de Referência em Saúde do Trabalhador (CEREST, Occupational Health Reference Centers) and the Vigilância em Saúde do Trabalhador (VISAT, Occupational Health Surveillance) play an important role in preventing and promoting occupational health. Occupational health is characterized as a set of interdisciplinary and multidisciplinary practices aimed at healing, preventing, and promoting health.^4^ These practices involve technical, social, political, and educational expertise aimed at analyzing and preventing conditions which cause occupational diseases and hazards.^4^

The role of edutainment in health education can be seen as a very useful tool in occupational health initiatives. In this context, the so-called “serious games” - games with learning objectives - can be used in several scenarios, providing guidance to children and adults in health prevention and promotion.^5^

Therefore, this article aims to describe the development of an educational technology from edutainment, targeting the prevention of occupational diseases and the promotion of occupational health.

## METHODS

A literature review on workers’ health and occupational risks was carried out aiming to provide information on the development of an educational technology based on occupational health conditions. We divided the study design into the following stages: research, development, construction, and final product.

We conducted a literature review on occupational diseases and ludic games. The occupational diseases used in this study were based on the following list of occupational diseases and hazards of the Sistema de Informação de Agravos de Notificação (Sinan, Brazilian Notifiable Diseases Information System): noise-induced hearing loss (NIHL), dermatosis, repetitive strain injury/work-related musculoskeletal disorders (RSI/WMSDs), pneumoconiosis, exposure to biological material, mental disorders, occupational cancer, and exogenous poisoning (pesticides). Child labor, work-related voice disorder (WRVD), and COVID-19 have been added to this game. Child labor is still a persistent, serious problem in Brazil, despite laws banning it. The WRVD is a disorder that significantly affects a large and important category of professionals: teachers. COVID-19 was also added to the hazards for this game because it is a pandemic that has victimized and impacted the lives of many workers around the world.

We used each of these occupational hazards individually as a keyword in the advanced search of two important occupational health journals in Brazil: Revista Brasileira de Medicina do Trabalho (RBMT, Brazilian Journal of Occupational Medicine) and Revista Brasileira de Saúde Ocupacional (RBSO, Brazilian Journal of Occupational Health). Then, articles have been chosen for the theoretical framework about these occupational hazards.

In the development stage, we analyzed what type of game would be designed, how it would be constructed, and what information about occupational hazards would be available. In addition, the game had to be affordable, easily accessible, and of interest to the participant to play.

According to information about game design, a game is composed of elementary points such as mechanics, aesthetics, narrative, and technology.^5^ The mechanics is how the game works, the aesthetics is the audiovisual aspect, the narrative is the sequence of events in the game, and the technology represents the media.^5^

To be a low-cost game, it was constructed with a basic technological resource: a printed cardboard. The game mechanics was chosen to be a trail game. The trail game is composed of squares, and the player advances the game after rolling the dice. Some squares consist of special points that allow the player to move forward or backward on the trail.

In order to assist the aesthetics and narrative of the game, a software designed the layout of the trail game, and six characters were created. These characters were inspired by various workers from different professional categories. The physical and behavioral characteristics of the characters were based on multiethnic and multicultural aspects comprising the Brazilian population.

**Figure 1:**
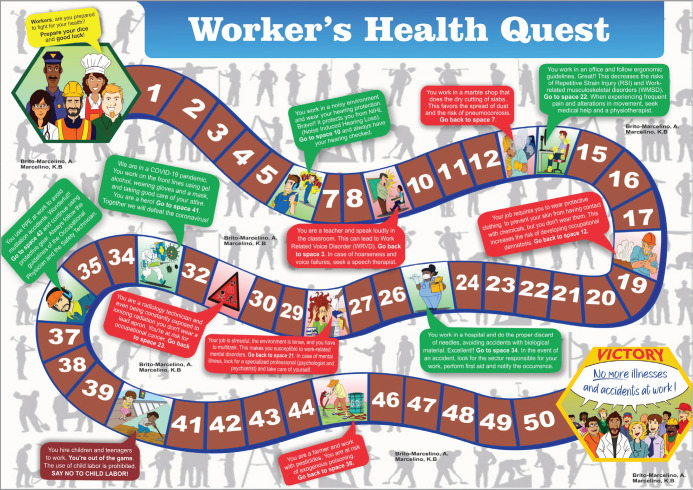
Trail game.

**Figure 2:**
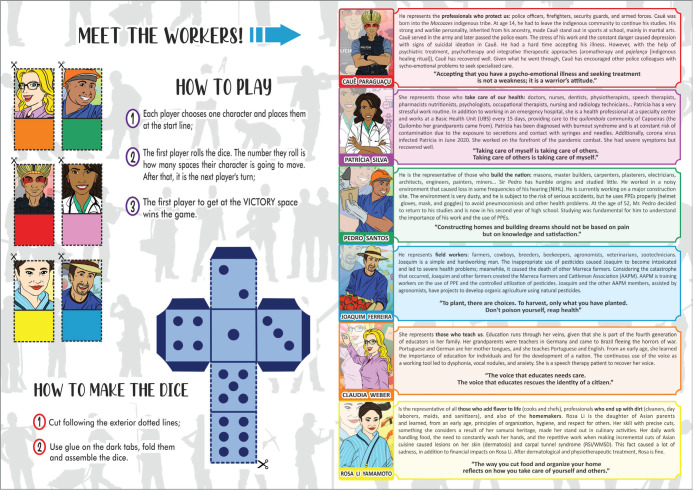
Instructions, dice, and characters.

To enhance the narrative gameplay, a short story was created for each character, showing their work history, their life story, and the occupational diseases they had to deal with. After all these steps, the game was completed, printed, and named (Figures 1 and 2).

## RESULTS

### THE GAME

Following literature review, a game was designed as a trail with 50 squares, 12 of which are special squares, containing diseases and risk factors for workers. The game was named “Trilhando pela saúde do trabalhador e da trabalhadora” (Worker’s Health Quest) (Figures 1 and 2). The game players included six characters representing professionals in the areas of education, health, construction, agriculture and cattle-raising, safety, hygiene, and food.

The literature review that supported this study and the creation of the edutainment trail game was based on 22 studies about occupational diseases.

The first disease represented in the game is NIHL, a common occupational disease in Brazil.^6^ It is a progressive, cumulative, insidious, and irreversible disease that compromises hearing.^7^

Continuous exposure to occupational noise causes hair cells of the cochlea to degenerate, leading to sensorineural and bilateral hearing loss, which initially affects the higher frequencies of hearing, then affects the other frequencies.^7^ Although NIHL is irreversible, there are a series of regulations and prevention standards, among which we highlight the mandatory use of hearing protectors and periodic hearing tests for workers exposed to noise.^6^

The second disease included was WRVD. Nearly one-third of professionals use their voice as a working tool.^8^ Teachers, singers, actors, telemarketing operators, announcers, pastors, and a number of other professionals use their voices as part of their profession.^9^ Thus, WRVD arises from occupational activities and inappropriate use of the phonation apparatus.^9^

WRVD is defined as a vocal alteration of slow and progressive evolution, with or without the presence of vocal fold lesions. Hoarseness, tiredness when speaking, shortness of breath, hawking, burning, throat dryness, and difficulty to project your voice are symptoms of this disorder. In addition to these symptoms, organic alterations in the laryngeal structures, such as nodules, clefts, cysts, edemas, and polyps can appear in professionals who use their voices strenuously and with no preventive care.^8^

Teachers are the professionals most commonly affected by WRVD.^8^ The continuous use of voice during class, the effort to project their voice, the lack of knowledge about vocal techniques, crowded classrooms, poor acoustics, and the sound competition produced by noisy students contribute to increase WRVD in educators.^8^

In addition, the absence of formal recognition of WRVD as an occupational disease makes it impossible to add this disorder to Sinan. This compromises the identification of ill workers and prevents the creation of public policies aimed at the promotion of vocal health.^9^

Pneumoconiosis was the third disease added to the game, and it is characterized by a lung disease associated with exposure to dust. This disease is often related to exposure to silica, coal, marble, and other substances in the workplace.^10^ Dust inhalation can cause symptoms such as progressive dyspnea, fever, cough, and weight loss.^11^ Professionals who are exposed to dust from mining, porcelain manufacturing, stone and marble cutting, production of water tanks and asbestos roof tiles, construction, sandblasting, and agriculture are susceptible to pneumoconiosis.^10^

Clinical signs and imaging tests (X-ray and CT scan) are of great importance to diagnose pneumoconiosis. Diffuse and bilateral micronodules and rings of lymph node calcification can be observed in imaging tests.^10^

Brazil has implemented the Programa Nacional de Eliminação da Silicose (PNES, National Program for the Elimination of Silicosis) since 2001, following the guidelines of the International Labor Organization and the World Health Organization.^11^ This program aims to reduce the incidence of this disease by 2015, and to eliminate it as a public health emergency by 2030.^11^ Pneumoconiosis is still part of the Brazilian scenario, in spite of these efforts.

RSI/WMSDs are diseases caused by excessive use of the musculoskeletal system combined with the absence of recovery time of these anatomical structures.^12^ Currently, especially in countries with emerging economies, RSI/WMSDs affect a number of professionals: bank clerks, supermarket cashiers, typists, musicians, telephone operators, artists, dentists, and assembly line workers, among others.^13^ Various symptoms can appear insidiously in various parts of the body, such as pain, paresthesia, heaviness, and fatigue.^12^

The Ministry of Labor of Brazil has identified a list of WMSDs: cervicalgia, bursitis, cuff tear syndrome, tendinitis, carpal tunnel syndrome, and ulnar tunnel syndrome, among others.^12^

Occupational dermatoses are characterized by alterations in the mucous membranes, skin and its appendages, directly or indirectly caused by agents present in the occupational environment^14^. Laboratory tests, such as skin tests, should be performed to identify the causative agent.^14^

The most important guidance in the face of occupational dermatoses is to remove the causative substance from the vicinity of the worker. The proper use of personal protective equipment (PPE), special clothing, and correct personal hygiene are also essential for the prevention of this disease.^14^

Occupational risk with biological material was the sixth hazard included. This hazard is related to spreading diseases through contact with blood and other potentially contaminated fluids.^15^ This is the context in which health care professionals work. Health professionals are a broad group of workers, such as physicians, nurses, dentists, physical therapists, speech therapists, psychologists, nutritionists, pharmacists, occupational therapists, biomedicine professionals, acupuncturists, nursing technicians, and radiology technicians, among others.

Needle-stick and other sharps injuries are extremely dangerous because they can transmit more than 20 types of pathogens, including hepatitis B and C viruses and HIV.^16^ It is estimated that about 3 million accidents with sharps occur annually in the world.^16^ The main causes attributed to sharps accidents in the health care environment are improper disposal of contaminated materials and unprotected manipulation of needles.^15^

In addition, a number of risk factors that worsen the exposure of workers to biological material include contact with infected material and patient body fluids. The inadequate use of PPE, poor working conditions, lack of training, work overload, and continuous misuse of PPE increase the risk of contamination in these professionals.^17^

Work-related mental disorders were the seventh condition added to the game. These disorders occur when work demands do not match the capabilities, resources, and needs of workers.^2^

Occupational stress can trigger psychiatric disorders, such as depression, anxiety, panic syndrome, and burnout syndrome, and even lead the worker to suicide.^18^ In this respect, professional categories such as health provision, education, and public safety are among the categories most prone to mental disorders.

Health care professionals play a role of great responsibility in hectic, precarious environments, high work demands, absence of career plans, low salaries, and situations of therapeutic failure, in addition to experiencing the pain and death of patients on a daily basis.^1,18^

Furthermore, security professionals have an ostensive constitutional mission, aiming at maintaining law and order and ensuring rights are protected.^19^ Police officers have to deal with conflicting relations, violence, and criminality to perform their duties. Anti-crime operations demand intense physical and mental wear, leading these professionals to symptoms of insomnia, headache, irritability, aggressiveness, demotivation, and depression.^19^

Occupational cancer was also added to the game as an occupational disease. It is estimated that 8% to 16% of cancers are due to occupational exposure.^20^ This accounts for 1.3 million deaths due to neoplasms in association with occupational risks worldwide.^21^ There are many carcinogenic agents including dust (silica, asbestos, and wood dust), metals (lead, cadmium, beryllium, nickel), solvents (benzene, trichloroethylene), ionizing and non-ionizing radiation, changes in the circadian cycle, and cosmic radiation.^20^

In view of the multifactorial nature of cancer, it is difficult to establish a causal link, especially in the face of occupational risk factors. Many of these factors add up, making it difficult to identify the causative factor.^20^

COVID-19 was also included in the game. It is a highly contagious disease, caused by SARS-CoV-2. Its emergence occurred in the city of Wuhan, in the People’s Republic of China, and it has spread worldwide, reaching pandemic status on March 11, 2020.^22^

Health care professionals were on the forefront, fighting against the COVID-19 pandemic. Thus, these workers were more vulnerable to infection than the rest of the population. The infection rate among health care workers is 7.3% *versus* 5% in the general population.^23^

The correct use of PPE is essential for workers fighting against COVID-19. The use of aprons, PFF2 masks, goggles, caps, gowns, and gloves is recommended.^22^ Moreover, proper PPE dressing and undressing is important to prevent contamination.^22^ Vaccination of health care teams is also a health practice to prevent infection with SARS-CoV-2. The immunization of frontline workers should be a priority, because it minimizes the surge of new variants with greater resistance and lethality.^23^

Child labor has been added to the game. More than a hazard, child labor is illegal, although still common in Brazil. Nearly 5 million children and adolescents are engaged in legally prohibited professional activities. Working in dangerous and unhealthy environments denounces a scenario of cruelty to the physical, mental, and social integrity of these children.^24^

The profile of these young workers is predominantly male (65.1%); 33.5% work 40 hours or more per week; 48.6% are unpaid; more than half of them use chemicals and tools; and most of them work in rural areas.^24^

Child labor is also responsible for school dropout. A large number of children drop out of school to dedicate themselves to occupational activity. This leads to increased illiteracy rates and shortage of skilled labor in the future, thus increasing underemployment.^24^

Exogenous intoxication was the last hazard added to the game. The indiscriminate use of pesticides in Brazil causes negative impacts on the environment, on the health of rural workers and professionals working in pest control, and on consumers of contaminated food.^25^

Acute intoxications caused by exposure to pesticides have immediate effects, with signs and symptoms of poisoning. However, late effects of exposure are difficult to establish due to the time elapsed from exposure and involvement in other exposures, causing diseases.^25^ Neuropathies, skin diseases, teratogenic and carcinogenic effects, hearing loss, endocrine abnormalities, erectile dysfunction, and infertility are examples of disorders caused by pesticide intoxication.^25^

## DISCUSSION

Serious games have been used for some time in a variety of contexts. This approach allows the player to be immersed in educational objectives in a “playful” context.^5^

Playing does not only symbolize the memory of something experienced, but it is also a creative act and a way to build new possibilities.^26^ Symbolic play enables us to participate in new situations and different contexts without actually experiencing these realities.^26^ Therefore, it is possible to enable the worker, exposed to some occupational risk, to experience a situation that has not happened yet, and this sensitizes them to seek self-care and prevention.

Game-based learning is also characterized by an active way of playing.^27^ This process occurs horizontally between the educator and the student. The playfulness allows the concepts and information to be acquired attractively and enjoyably.^27^

The development of new technology in the learning process is necessary in the health context.^5^ In health care, there are other experiences using trail games. Games about alcoholism prevention, information for pregnant women, *Aedes aegypti* control, and teacher’s vocal health care have been found in specialized literature.^28^

As for occupational health, the game “Trilhando pela saúde vocal do professor” (Teachers’ vocal health quest) presented a methodology similar to the one described in this study. The care and prevention of WRVDs, especially in professionals who use their voice as a tool to teach, have great social importance.^28^

The choice of using a trail game as an interface for edutainment was based on criteria such as low cost, interactivity, and easy understanding about occupational health hazards.

This study, on the other hand, has limitations, especially regarding the impossibility of workers’ feedback. The absence of the target audience’s opinion and the impossibility of measuring the information the audience has learned has limited this study.

Despite the limited use of board games in health care, studies show that the results in the dynamics of education and learning are positive.^27^

## CONCLUSIONS

Edutainment is considered an important tool to develop learning, promotion, and leisure processes. The trail game, containing information about diseases, occupational health, and the care that workers should take in their work environment, contributes to the prevention of occupational diseases.

These games can be implemented in several occupational environments, as simple and low-cost alternatives to promote workers’ awareness and to minimize occupational hazards.
